# Formation of Stable Vascular Networks by 3D Coaxial Printing and Schiff-Based Reaction

**DOI:** 10.3390/gels10060366

**Published:** 2024-05-25

**Authors:** Jingxin Shan, Zhiyuan Kong, Xiaohong Wang

**Affiliations:** 1 Center of 3D Printing & Organ Manufacturing, School of Intelligent Medicine, China Medical University (CMU), No. 77 Puhe Road, Shenyang North New Area, Shenyang 110122, China; shanjingxin@huh.edu.cn (J.S.); 2020121772@stu.cmu.edu.cn (Z.K.); 2 Department of Biomedical Engineering, He University, Shenyang 110163, China; 3 Guangdong Key Lab of Orthopedic Technology and Implant Materials, General Hospital of Southern Theater Command of PLA, Guangzhou 510010, China; 4 Key Laboratory for Advanced Materials Processing Technology, Ministry of Education & Center of Organ Manufacturing, Department of Mechanical Engineering, Tsinghua University, Beijing 100084, China

**Keywords:** 3D bioprinting, oxidized polysaccharides, double crosslinked gelatin–alginate hydrogels, coaxial 3D printing, vascular network

## Abstract

Vascularized organs hold potential for various applications, such as organ transplantation, drug screening, and pathological model establishment. Nevertheless, the in vitro construction of such organs encounters many challenges, including the incorporation of intricate vascular networks, the regulation of blood vessel connectivity, and the degree of endothelialization within the inner cavities. Natural polymeric hydrogels, such as gelatin and alginate, have been widely used in three-dimensional (3D) bioprinting since 2005. However, a significant disparity exists between the mechanical properties of the hydrogel materials and those of human soft tissues, necessitating the enhancement of their mechanical properties through modifications or crosslinking. In this study, we aim to enhance the structural stability of gelatin–alginate hydrogels by crosslinking gelatin molecules with oxidized pullulan (i.e., a polysaccharide) and alginate molecules with calcium chloride (CaCl_2_). A continuous small-diameter vascular network with an average outer diameter of 1 mm and an endothelialized inner surface is constructed by printing the cell-laden hydrogels as bioinks using a coaxial 3D bioprinter. The findings demonstrate that the single oxidized pullulan crosslinked gelatin and oxidized pullulan/CaCl_2_ double-crosslinked gelatin–alginate hydrogels both exhibit a superior structural stability compared to their origins and CaCl_2_ solely crosslinked gelatin–alginate hydrogels. Moreover, the innovative gelatin and gelatin–alginate hydrogels, which have excellent biocompatibilities and very low prices compared with other hydrogels, can be used directly for tissue/organ construction, tissue/organ repairment, and cell/drug transportation.

## 1. Introduction

For a long time, the in vitro building of tissues/organs has been a goal in biomaterials, tissue engineering, and regenerative medicine [1]. However, angiogenesis has always been a challenging issue that hinders organ construction and regeneration. Tissues thicker than 1 mm struggle to maintain normal vitality without vascularization, as the diffusion limit of nutrients/oxygen is approximately 100–200 μm [2,3].

So far, few studies have reported the successful construction of bioartificial blood vessels, as well as vascular networks, in vitro. For example, Bell et al. assembled a blood vessel using smooth muscle cells, endothelial cells, and collagen in 1986 [4]. Yan et al. synthesized a polyurethane (PU)/heparin vascular graft for small-caliber vein repair in 2007 [5]. Xu et al. used three-dimensional (3D)-printed PU for vascular network building in 2008 [6]. Kirkton et al. designed a blood vessel that entered the clinical trial phase in 2019 [7]. Nevertheless, when these blood vessels or vascular networks are used for in vivo implantation, they encounter a lot of bottleneck problems, such as an unmatched size and composition, a lack of endothelialized vascular networks, and the appearance of side reactions (e.g., thrombus and tissue necrosis). Consequently, there is an immediate requirement for a structural framework that can encompass the complete vascular hierarchy with the aim of facilitating organ repair and replacement.

Since 2005, 3D printing has been widely applied in the fields of tissue engineering, regenerative medicine, and organ manufacturing, providing a building method that enables the precise control of the 3D structures and cell arrangements in an object or construct [8,9,10,11]. Traditional 3D bioprinting technologies include extrusion-based 3D printing, inkjet-based 3D printing, and laser-assisted 3D printing [11,12]. Among them, extrusion-based 3D printing is currently the most widely used technology due to its ease of use, fast formation, and low cost [13]. However, the resolution of squeeze-out printing is relatively low, and it is susceptible to external conditions. Recently, a special type of 3D printing, i.e., coaxial printing, has been explored. Compared to the single nozzle used in traditional extrusion-based 3D bioprinting, the nested coaxial nozzles provide more possibility and creativity [14]. Two different kinds of living cells can be encapsulated in the outer and inner material barrels and 3D printed simultaneously, mimicking the arrangement of the cells in a natural blood vessel [15].

An essential concern of 3D bioprinting pertains to the capability to consistently generate replicable 3D constructs using bioinks, a category of biomaterials that can encapsulate living cells and bioactive agents. In order to address this objective, hydrogels have gained significant popularity due to their biocompatible milieu and substantial water content. Hydrogels are 3D crosslinked networks of hydrophilic polymers, which are usually soft with a high water content, like the extracellular matrix (ECM) [16]. Natural hydrogels, such as gelatin and alginate, have played key roles in cell activities, including proliferation, migration, and lineage-specific gene expression [17,18,19]. The interconnected porous structures of the polymeric networks enable the transport of oxygen, nutrients, and metabolites for the encapsulated cells [20].

Gelatin is a water-soluble protein derived from the partial hydrolysis of the natural polymer collagen with very special properties [21]. Gelatin solution can form hydrogel via hydrogen bond formation in or among the polymeric molecules below 25 °C, which is a physical crosslinking process. This physical crosslinking is unstable and reversible. When the temperature reaches the phase transition temperature of 25 °C, the gel-state hydrogels return to sol-state solutions again. Considering this feature, gelatin hydrogels can be 3D printed layer-by-layer between 1 and 20 °C, but the poor thermostability of gelatin hydrogels has greatly limited their applications in physiological conditions, such as 37 °C [22]. The chemical or biochemical crosslinking of gelatin molecules has often been used after 3D printing to enhance the structural stability of the 3D-printed constructs. The commonly used crosslinking agents include aldehydes (such as glutaraldehyde [23] or glyceraldehyde [24]), polyoxides [25], 1-ethyl-3-(3-Dimethylaminopropyl)-carbodiimide [26], transglutaminase [27], and genipin [28]. However, most of these crosslinking agents exhibit cytotoxicity, a high cost, and result in an excessive darkening of color post-crosslinking. In particular, a critical drawback arises when the gelatin molecules are crosslinked with aldehyde agents like glutaraldehyde and glyceraldehyde, as their degradation products can release toxic substances [23].

In recent years, oxidized polysaccharides have been recognized as effective crosslinking reagents for amino-containing polymers. The oxidized polysaccharide crosslinking agent exhibits a favorable biocompatibility, with degradation products that are non-toxic [29]. Furthermore, crosslinked gelatin molecules with modified polysaccharides are beneficial for simulating the main components (i.e., proteoglycan) of the ECM and increasing the stability of hydrogels [30]. Polysaccharides acquire aldehyde groups via oxidation, leading to the formation of covalent crosslinks between the aldehyde groups and the free amino groups of lysine and hydroxylysine in gelatin molecules through the Schiff base reaction mechanism [31,32].

In this study, we utilized oxidized pullulan (OxP) to crosslink gelatin molecules, with the objective of extending their degradation rate. To achieve this, we created a dual crosslinked gelatin–alginate hydrogel by combining gelatin with sodium alginate solutions, aiming to mimic the ECM from various angles. 3D coaxial bioprinting technology was effectively created to produce a vascular network, followed by the successful establishment of a vascularized organ model around the vascular network.

## 2. Results

### 2.1. Characterization of the OxP Crosslinked Gelatin Hydrogels

**Figure 1:**
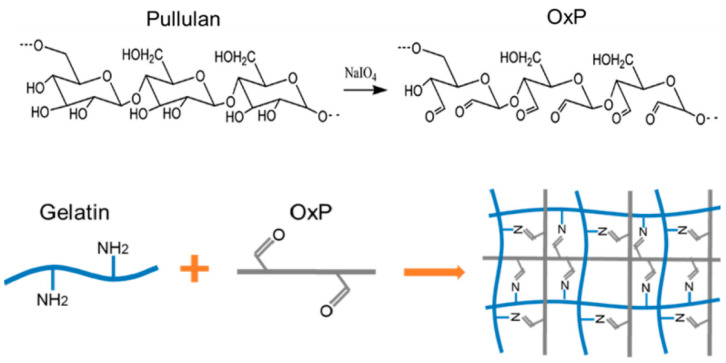
Schematic diagram of the OxP crosslinked gelatin molecules based on Schiff base reaction.

The crosslinking mechanism of the gelatin molecules with OxP is shown in Figure 1. Basically, there are a lot of hydroxyl (-OH) groups in the pullulan molecules. When these -OH groups are oxidized by sodium periodate (NaIO_4_) under acid conditions, the pullulan molecules are changed into OxP molecules with a lot of aldehyde (-CHO) groups. The -CHO groups in the OxP molecules then react with the amino (-NH_2_) in the gelatin molecules based on the Schiff base reaction under mild conditions, such as at 37 °C and pH 4.0.

**Figure 2:**
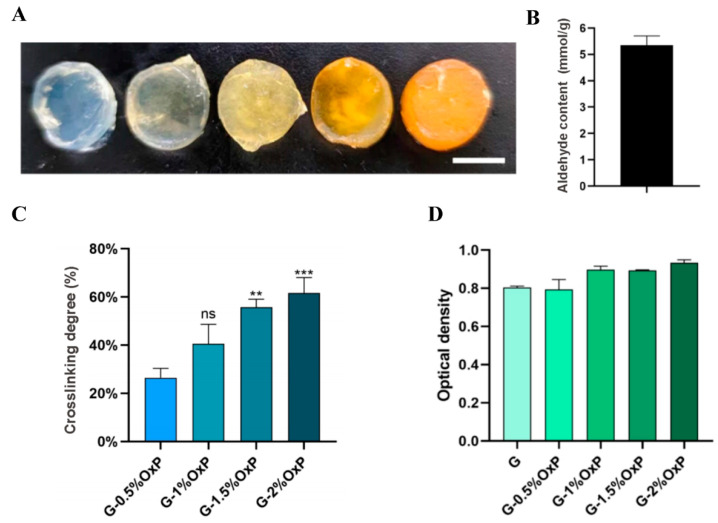
Characterization of the OxP crosslinked gelatin hydrogels. (**A**) The molding results of the OxP crosslinked gelatin hydrogels with different concentrations of OxP. Scale bar = 1 cm. (**B**) The oxidized result of the pullulan. (**C**) Crosslinking degrees of the OxP crosslinked gelatin hydrogels with different concentrations of OxP measured through ninhydrin method (N = 3). **: *p* < 0.01, ***: *p* < 0.001 vs. G-0.5% OxP. (**D**) Cytotoxicity of the OxP crosslinked gelatin hydrogels (N = 3).

In Figure 2A, the color transition of the OxP crosslinked gelatin hydrogels from white to yellow with increased OxP concentrations (i.e., 0, 0.5, 1, 1.5, and 2% *w*/*v*) is manifested. This phenomenon is associated with the formation of C=N bonds between the aldehydes and amino groups in the gelatin molecules [23]. The amount of aldehyde groups obtained after reacting for 4 h at pH 4 and room temperature was measured by NaOH titration to be 5.37 mmol/g (Figure 2B).

The OxP crosslinking degree is shown in Figure 2C. It is noteworthy that, as the concentration of OxP increased, there was an observed upward trend in the degree of crosslinking. More specifically, the crosslinking degrees for 0.5%, 1%, 1.5%, and 2% OxP were measured as 26.4%, 40.6%, 55.7%, and 61.6%, respectively. Even when the OxP concentration increased from 1.5% to 2%, the crosslinking degree remained relatively steady at approximately 60%. The changes in the crosslinking degree aligned with the color gradients of the hydrogels (Figure 2A) achieved through the partial crosslinking. 

In terms of the cytotoxicity assessment, the survival ratio of the cells encapsulated in the hydrogels in each experimental group remained above 80% following a 24 h culturing period (Figure 2D). The findings demonstrated that the prepared OxP exhibited minimal residual NaIO_4_, and the OxP crosslinked gelatin hydrogels displayed nearly non-toxicity towards cells, thereby indicating a high level of biocompatibility.

### 2.2. Characterization of the Double-Crosslinked Gelatin–Alginate Hydrogels

**Figure 3:**
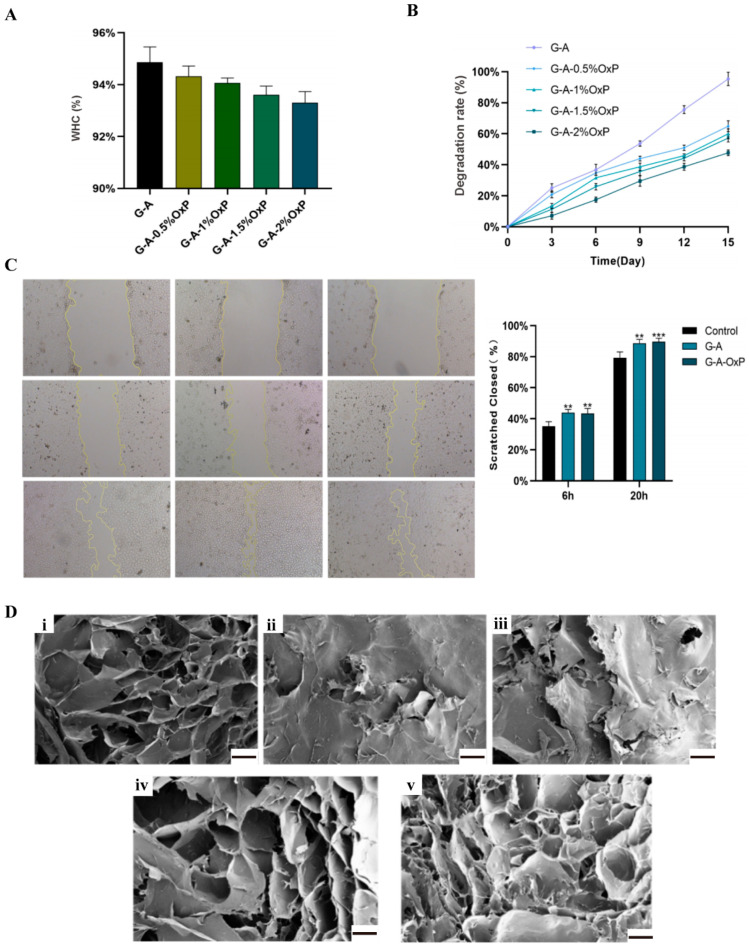
Characterization of the OxP and CaCl_2_ double-crosslinked gelatin-alginate hydrogels. (**A**) Water-holding capacity (WHC) of the gelatin–alginate hydrogels (N = 3). (**B**) Degradation rates of the of the gelatin–alginate hydrogels (N = 3). (**C**) Cell migration capabilities (0 h, 6 h, and 20 h) after culturing with the hydrogel extracts, expressed as mean ± standard deviation (N = 3); one-way analysis of variance was used for comparison between groups; **: *p* < 0.01, ***: *p* < 0.001 vs. Control. (**D**) Micropore structures in the hydrogels after being dehydrated (N = 3). (**i**) G-A; (**ii**) G-A-0.5% OxP; (**iii**) G-A-1% OxP; (**iv**) G-A-1.5% OxP; and (**v**) G-A-2% OxP. Scale bare = 200 μm.

From Figure 3A, it can be seen that there was a decreasing trend in the water-holding capacity (WHC) of the gelatin–alginate hydrogels with an increase in the concentration of OxP. This is because, with the augmentation of the OxP concentration, a more compacted polymeric structure with more crosslinks was produced, leading to a reduction in the WHC. Nevertheless, when the concentration of the OxP reached 2%, the value of the WHC was still more than 93%, meaning that the hydrogel could retain a substantial capacity for liquid absorption.

As for the degradation rates of the OxP and CaCl_2_ double-crosslinked gelatin–alginate hydrogels, it was evident that all the double-crosslinked hydrogels exhibited a reduced degradation rate compared to the CaCl_2_ single-crosslinked hydrogels (Figure 3B). On day 15, the degradation rate of the CaCl_2_ single-crosslinked gelatin–alginate hydrogel exceeded 90%, whereas each of the OxP and CaCl_2_ double-crosslinked gelatin–alginate hydrogels remained below or approximately 60%.

The migratory capacity of human umbilical vein endothelial cells (HUVECs) within the double-crosslinked hydrogels was assessed using a cell scratching technique. In both the groups treated with the single-crosslinked and double-crosslinked gelatin–alginate hydrogel extracts, the closure of the scratch exceeded 40% after 6 h and approximately 90% after 20 h, demonstrating a superior efficacy compared to the control group (Figure 3C).

The microstructures of the double-crosslinked hydrogels via different concentrations of OxP and dehydration processes are shown in Figure 3D. In the double-crosslinked hydrogels at the concentrations of 0.5% and 1% OxP, the pores were less and uneven (Figure 3D(ii,iii)). At the concentrations of 1.5% and 2%, the pore numbers and sizes were significantly increased and the wall thickness was more uniform (Figure 3D(iv,v)).

Based on the performance of the test, we selected G-A-2% OxP for the subsequent 3D printing of the vascular networks.

### 2.3. Induction and Identification of the Adipose-Derived Stem Cells (ASCs)

**Figure 4:**
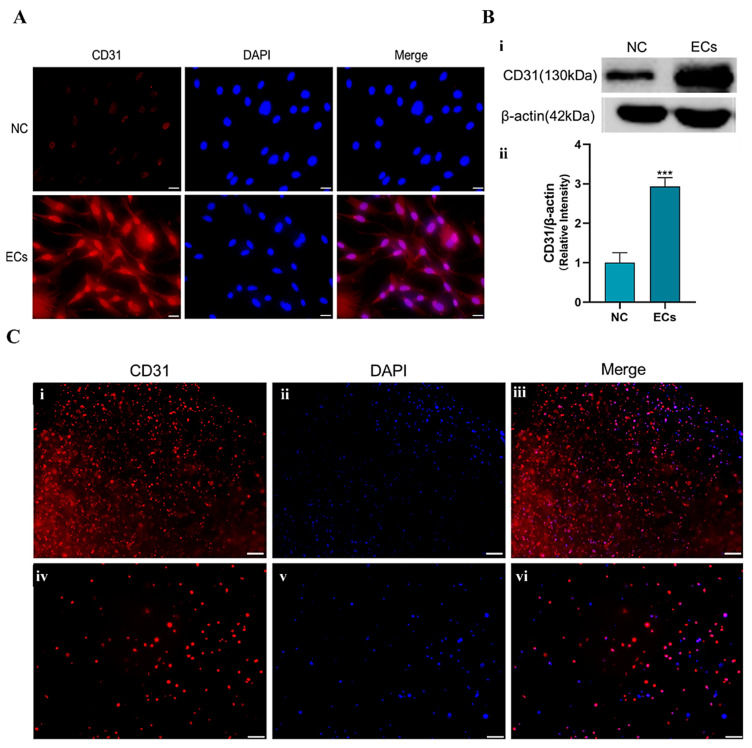
(**A**) Immunofluorescence results of the induced differentiation of the adipose-derived stem cells (ASCs) into endothelial cells, scale bar = 20 μm. Red: AlexaFluro555 staining the endothelial cell mark CD31 with red color, meaning that the ASCs had differentiated into endothelial cells; Blue: 4′,6-diamidino-2-phenylindole (DAPI) staining for the living cell nuclei, suggesting that the cells were alive. (**B**) (**i**) Western Blot results of the induced endothelial cell marks (i.e., CD31 and ß-actin) in the control group (i.e., NC) and experimental group (i.e., ECs). (**ii**) ***: *p* < 0.001. (**C**) Immunofluorescence staining of the endothelial cells differentiated from the ASCs in the double crosslinked gelatin-alginate hydrogel on day 7. Scale bar: (**i**–**iii**) = 200 μm; (**iv**–**vi**) = 100 μm.

Figure 4A shows the immunofluorescence staining results of the ASCs after 7 days of the growth factor induction. It can be seen that the expression of CD 31 was positive in the endothelial growth factor induction group (i.e., ECs), while there was basically no render in the no growth factor induction control group (i.e., NC). These results can also be reflected by the gel electrophoresis experiments, in which the CD 31 (i.e., protein) expression in the induced group was significantly higher than that in the uninduced group, indicating that the ASCs were successfully induced into endothelial cells, as shown in Figure 4B.

Figure 4C shows the endothelialization induction results of the ASCs encapsulated in the double-crosslinked gelatin–alginate hydrogels after 7 days’ induction using the growth-factor-incorporated medium. After immunofluorescence staining, it was found that the expression of endothelial cell marker CD 31 was positive, indicating that the ASCs were successfully induced into endothelial cells within the double-crosslinked gelatin–alginate hydrogel.

### 2.4. Printability of the Single- or Double-Crosslinked Hydrogels

**Figure 5:**
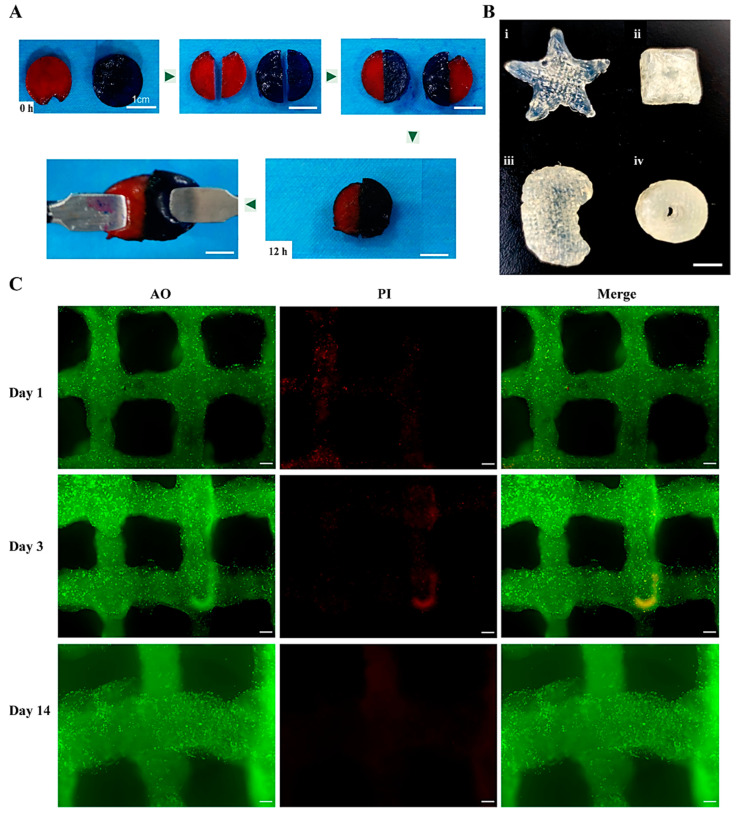
Performance of the single- and double-crosslinked hydrogels. (**A**) Self-healing capacity of the OxP crosslinked gelatin hydrogel. Scale bar = 1 cm. In order to obtain a good visual effect, two pigments (i.e., red and blue) were added to the OxP crosslinked disc-shaped gelatin hydrogels before they were cut and re-organized. (**B**) The 3D-printed complex structures using the double-crosslinked gelatin–alginate hydrogels as bioinks. (**i**–**iv**) represent the pentagram, cuboid, kidney, and hollow cylinder models, respectively. Scale bar = 1 cm. (**C**) Cell compatibility in the 3D printed gelatin–alginate hydrogels (AO/PI staining, AO: Acridine orange, green; PI: Propidium iodide, red). Scale bar = 200 μm. Bright green means that the cells were in living states, while red means that the cells were in dead states.

In Figure 5A, the blurring of the restructured boundary of the OxP crosslinked gelatin hydrogel can be observed after a duration of 12 h. When a tweezer was used to stretch the restructured gel, the hydrogel remained stable with the applied stretching force, thereby indicating its cohesive healing properties. Figure 5B demonstrates the 3D-printed molds with the OxP and CaCl_2_ double-crosslinked gelatin–alginate hydrogels (i.e., 5% gelatin—3% and sodium alginate—2% OxP). For the stellar and cuboid models, 15 layers of the double-crosslinked gelatin–alginate hydrogel were printed (Figure 5B(i,ii)). For the kidney-like and hollow cylinder models, more than 40 layers of the double-crosslinked gelatin–alginate hydrogel were printed (Figure 5B(iii,iv)). These results indicate that the OxP single-crosslinked gelatin and the OxP and CaCl_2_ double-crosslinked gelatin–alginate hydrogels both had stable polymeric networks for 3D printing or construction. 

Figure 5C depicts the staining outcomes of the 3D-printed cell-laden grid constructs after 1, 3, and 14 days of cultivation. The 3D-printed grid structures were stained as green, meanwhile, the living cells in the hydrogel were stained as bright green and the dead cells were stained as red. Initially, on day 1, most of the cells exhibited a green hue, while a fraction displayed a red tint. Evidently, the cell numbers increased prominently on day 3, which obviously surpassed those on day 1. The red color declined sharply, signifying the cells’ acclimation to the hydrogel microenvironment and that their proliferation capabilities had been activated. On day 14, the densities of the cells were further enhanced with nearly no red color, whereas the grid structures remained very well. 

### 2.5. Characterization of the Coaxial 3D-Printed Vascular Networks

**Figure 6:**
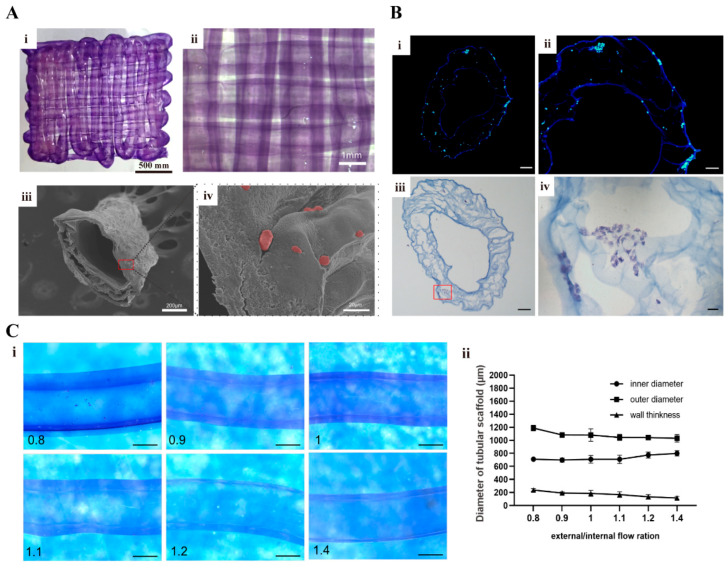
Characterization of the 3D-printed vascular networks. (**A**) (**i**) Confocal microscope image of a tubular vascular network without cells. (**ii**) The magnified view of (**i**). (**iii**) Microstructure of the 3D-printed vascular network after dehydration. (**iv**) The cells adhered to the outer surface of the vascular network after being dehydrated and stained. (**B**) (**i**,**iii**) represent the DAPI and HE staining results of the 3D-printed vascular network. (**ii**,**iv**) represent the enlarged view of (**i**,**iii**), respectively. (**C**) (**i**) Visual image of the tubular network with different flow rates (outer/inner axis) under stereo microscope. Scare bar = 500 μm. (**ii**) Statistical graph for diameter of tubular scaffold.

To enhance the elucidation effects of the hollow architecture, a minute quantity of hematoxylin dye was incorporated into the hydrogel, resulting in a discernible purple hue of the 3D-printed tubular vascular networks (Figure 6A(i,ii)). The inner and outer boundaries of the tubular structures were very clear to the naked eye. After dehydrating, the cell-laden hydrogel became porous, with a lot of micropores in the middle of the hydrogel layer (Figure 6A(iii)). These micropores were favorable for cell accommodation and mass exchange. The red hue depicted in Figure 6A(iv) illustrates some of the cell states post-staining, and these cells still adhered to the external surface of the vascular networks. Figure 6B shows the 4′,6-Diamidino-2-phenylindole (DAPI) and hematoxylin-eosin (HE) staining pictures of the 3D-printed tubular vascular networks after 3 d of in vitro culture. The bracket in Figure 6B(i) exhibits a ring-shaped structure with a relatively smooth surface. Additionally, Figure 6B(iii) reveals the aggregation of the encapsulated cells into clumps and their tendency to form tubes, suggesting favorable growth of the endothelial cells within the tubular vascular networks.

Figure 6C demonstrates the relationships between the different inner/outer axial flow velocities and the diameters of the 3D-printed tubular vascular networks. It was found that an increase in the flow rate of the outer axis led to a decrease in the outer diameter of the vascular networks, resulting in a reduction in the tubular wall thickness. When the ratio of the outer to inner flow rate was 1.4, the average tubular wall thickness was measured as 238 μm. Conversely, when this ratio decreased to 0.8, the average tubular wall thickness diminished to 116 μm. The connectivity and penetration of the vascular networks may be compromised if the tubular wall is excessively thick, whereas the supportive performance of the vascular networks declines significantly if the tubular wall is excessively thin. Consequently, selecting a proper external to internal flow rate ratio is vitally important for vascular network construction. In our experiments, approximately 1 was chosen as the suitable 3D printing parameter.

### 2.6. The Coaxial 3D-Printed Vascular Tissue and Connectivity of the Vascular Network

**Figure 7:**
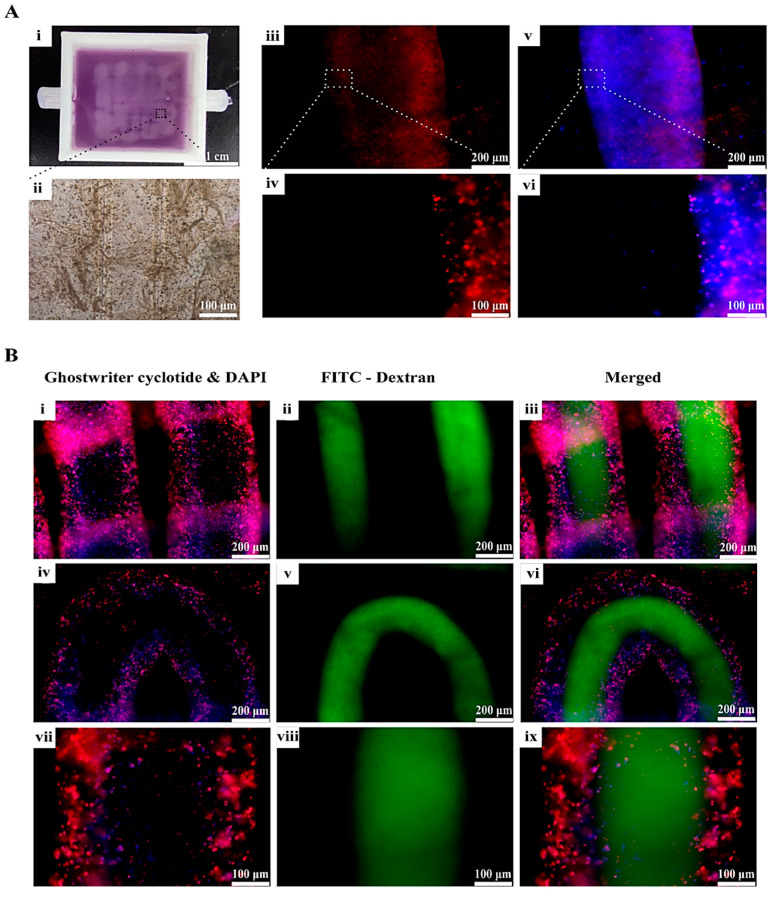
A coaxial 3D-printed vascular tissue and the connectivity of the vascular networks. (**A**) (**i**) Physical diagram of a coaxial 3D-printed vascularized tissue. (**ii**) The vascularized tissue under light microscope. (**iii**) A CD31 immunofluorescence picture with low magnification. (**iv**) Enlarged picture of (**iii**). (**v**) CD 31 merged with DAPI. (**vi**) Enlarged picture of (**v**). (**B**) A corner of the grid vascular network. (**i**,**iv**,**vii**) represent the staining results of the phalloidin (AbFluor 594, red) and DAPI fluorescence (blue). (**ii**,**v**,**viii**) represent the inside injected FITC-dextran (green). (**iii**,**vi**,**ix**) represent the merged results.

In Figure 7A, a coaxial 3D-printed vascular tissue is displayed using the double-crosslinked hydrogel as bioink. The smooth and uniform appearance of the 3D-printed vascular network suggests that this 3D printing method is effective in constructing vascularized tissues. HUVECs remained robust following a 7-day period of in vitro culturing in the 3D-printed hydrogels. 

Phalloidin staining revealed the formation of clumps among the HUVECs within the tubular vascular network after 7 days of culture (Figure 7B). Upon infusion of the FITC-labeled dextran solution, no instances of leakage were observed in either the straight part (Figure 7B(iii)) or corner part (Figure 7B(vi)) of the tubular vascular network. Upon closer examination, it became evident that the dextran solution did not promptly infiltrate the tubular wall, but rather distributed uniformly within the inner cavity (Figure 7B(ix)). This observation underscores the favorable connectivity of the 3D-printed tubular vascular network and the gradual permeation of the solution within the tubular structures, thereby holding substantial implications for the subsequent perfusion culture and blood vessel maturation endeavors.

## 3. Discussion

There are several indexes that are very important for hydrogel biomaterials. WHC refers to the capacity of a hydrogel to retain water in the presence of gravitational forces. This characteristic serves as a significant physical and chemical indicator for hydrogel materials. Simultaneously, an appropriate degradation rate of the polymers is also a crucial attribute for hydrogels, as it ensures the maintenance of a stable environment and the provision of adequate growth space for the enclosed cells. When the commonly utilized CaCl_2_ single-crosslinked gelatin–alginate hydrogel was subjected to a 37 °C culture after 3D printing, the gelatin molecules dissolved or degraded rapidly, resulting in the loss of certain cells and instability of the 3D constructs. However, by crosslinking the gelatin molecules with OxP, the structural stability can be significantly enhanced. An increase in the OxP concentration led to a relative decrease in the degradation rate. This phenomenon can be attributed to the fact that as the degree of OxP crosslinking in the gelatin molecules increased, the overall 3D structure became more compact or tight.

In order to solve the unstable problems of the physical crosslinked gelatin based hydrogels, biocompatible OxP was chosen as the chemical crosslinking agent. OxP was chosen due to its non-toxic nature, anti-bacterial properties, and minimal impact on color alteration, distinguishing it from other alternative crosslinking agents [33,34,35]. A set of tests were carried out on the single- and double-crosslinked gelatin and gelatin–alginate hydrogels with varying concentrations of OxP. The results demonstrated that the hydrogels crosslinked with 2% (*w*/*v*) OxP exhibited a superior degradation persistence and structural stability compared to the physcial crosslinked gelatin hydrogels. When these hydrogels were 3D printed with a coaxial nozzle, a vascular network with an outer diameter of approximately 1 mm was effectively created, while tubular vascular networks with varying inner diameters and wall thicknesses were achieved by optimizing the extrusion parameters. These findings hold substantial implications for the advancement of complex tissue and organ engineering within the fields of biomaterials, tissue engineering, organ manufacturing, and regenerative medicine.

As stated above, vascularization plays a pivotal role in the successful construction of thick-volume organs for organ transplantation. However, it currently faces many challenges. A novel approach in bioprinting known as coaxial 3D bioprinting has emerged [36]. This technique involves the simultaneous extrusion of two distinct bioink formulas in a core–shell configuration, enabling the direct formation of bionic vascular structures. The creation of a small-diameter tubular structure, averaging several hundred microns, can be achieved through regulating the inner and outer diameters of the coaxial nozzles or needles. Concurrently, the incorporation of endothelial cells within this structure offers a viable approach to facilitate channel endothelialization. In comparison to some other bioprinting technologies, such as single-nozzle inkjet-bioprinting, coaxial 3D printing has the capability to simultaneously print multiple functionalized biomaterials, thereby establishing a basis for the construction of intricate vascular tissues or organs [37]. In contrast to photo-crosslinking printing, coaxial printing eliminates the need for extra crosslinking techniques involving ultraviolet light and potential cytotoxic agents [38].

Alginate solution or hydrogel is the most used bioink in coaxial printing due to its rapid crosslinking properties with Ca^2+^ [39] The alginate bioink and crosslinking CaCl_2_ solution can be simultaneously extruded through the coaxial nozzle, resulting in immediate gelation within the dispensing head, followed by the direct formation of a hollow tubular structure. Consequently, when the double-crosslinked gelatin–alginate hydrogel was printed with the coaxial technique, the construction process of the endothelialized vascular networks was greatly facilitated with an enhanced structural stability. In forthcoming research, the manipulation of parameters such as the coaxial needle size, in conjunction with the integration of additional printing technologies, holds promise for the fabrication of full-size, multi-scale functionalized vascular networks. Through the development of intricate systems and the incorporation of various stromal cells, including but not limited to cancer cells, liver cells, and fibroblasts, the in vitro creation of vascularized tissues and organs that closely mimic their natural counterparts of the human body can be achieved.

There are several limitations that exist in this study. Primarily, our focus was on enhancing the biocompatibility, degradability, and printability of the gelatin based hydrogels through double-crosslinking modifications. However, due to the lack of an equivalent, we did not conduct any tests on their mechanical performances, such as tensile strength and torsional resistance. Future endeavors should prioritize the improvement of hydrogels’ mechanical strength and the reinforcement of vascular networks to withstand the filling pressure generated during blood flow. Moreover, the inherent vulnerability of the printed vascular networks, characterized by their thin wall thickness of approximately 200 μm, renders them susceptible to collapse and compromised structural integrity when subjected to prolonged in vitro cultures without supplementary reinforcement. It is hoped that the refinement of the coaxial printing structures can be achieved through incorporating additional biomaterials for the construction of a vascular smooth muscle layer, extending beyond the endothelial layer. Furthermore, the rheological properties of bioinks after OxP crosslinking are hard to define. Subsequent studies may need to integrate different printing technologies with novel bioinks to produce functionalized vascular networks with fully sized and multi-tissue characteristics.

## 4. Conclusions

Utilizing the Schiff base reaction mechanism, we employed oxidized polysaccharide to crosslink gelatin molecules in order to create stable gelatin and gelatin–alginate hydrogels. The OxP crosslinked gelatin-based hydrogels were more stable than their origins, with delayed degradation rates. Using coaxial 3D bioprinting technology, we successfully constructed a continuous vascular network with HUVECs embedded in the OxP and CaCl_2_ double-crosslinked gelatin–alginate hydrogels. Vascularized cancer tissue was then created using the cell-laden hydrogels and coaxial 3D bioprinting technology. These achievements hold considerable implications for the automatic manufacturing of complex bioartificial organs in vitro. Further utilization of these techniques with improved printing accuracies and vascularized networks will be exploited and reported. 

## 5. Materials and Methods

### 5.1. Synthesis of OxP and the OxP Crosslinked Gelatin Based Hydrogels

The synthesis process of the OxP referred to the report by Spatareanu and colleagues [40]. Briefly, a dilute H_2_SO_4_ solution with a pH of 4.0, at a concentration of 100 mM, was prepared. In total, 2 g of pullulan was weighed and introduced into 70 mL of the diluted acid solution. The mixture was stirred in a dark environment for a duration of 30 min until complete dissolution occurred. Following this, 1.6 g of NaIO_4_ was weighed and dissolved in 30 mL of the dilute acid solution, also under dark conditions. The resulting NaIO_4_ solution was then added to the pullulan solution and stirred for 4 h.

In our previous study, a TG and CaCl_2_ double-crosslinked gelatin–alginate hydrogel, consisting of 5% gelatin and 3% sodium alginate, was formed, with excellent physical, chemical, and biological properties such as water retention, degradation, and biocompatibility [41]. In this study, our primary goal was to examine the influence of OxP on the gelatin based hydrogels. Consequently, we solely altered the ratio of OxP to gelatin within the OxP and CaCl_2_ double-crosslinked gelatin–alginate hydrogels, while maintaining the concentrations of gelatin and sodium alginate at 5% and 3%, respectively, as indicated in Table 1.

For measuring the crosslinking degree of the OxP crosslinked gelatin molecules, specific amounts of gelatin and OxP were weighed and dissolved in water. The resulting mixture was then subjected to a water bath at a temperature of 65 °C for a duration of 30 min. Once the gelatin was dissolved completely, the mixture was cooled to approximately 40 °C. Subsequently, the gelatin and OxP solutions were mixed thoroughly and allowed to react at a temperature of 37 °C for a period of 12 h.

The crosslinking degree of the gelatin molecules by OxP at various concentrations was assessed using the ninhydrin method, which is usually used to quantitatively measure free amino groups in samples. Simply, the OxP crosslinked hydrogel was mixed with 2 mL of deionized water and 1 mL of a 2% ninhydrin solution, heated in a water bath at 100 °C for 15 min, and then cooled. The quantification of free amino groups was carried out through spectrophotometry at a wavelength of 570 nm. The crosslinking degree was determined by employing the following calculation formula, as demonstrated by OD_d._

ODd=1−(ODsODnc)×100%

where OD*_S_* is the mole fraction of the free amino groups in the OxP crosslinked gelatin hydrogel and OD*_nc_* is the mole fraction of the free amino groups in the pure gelatin hydrogel.

### 5.2. Pullulan Oxidation Degree Measurement

The reaction between the amino group of hydroxylamine hydrochloride and the aldehyde group resulted in the formation of oxime and the release of HCl. Consequently, the concentration of aldehyde groups was determined by titrating the released HCl with NaOH, providing insight into the oxidation degree of the pullulan.

To determine the oxidation degree of the pullulan, 0.3 g of pullulan and OxP were dissolved in 15 mL of deionized water, respectively. This was followed by adding NaOH solution drop-by-drop while monitoring the pH with a meter until it reached 5.0. Then, 12 mL of a hydroxylamine hydrochloride solution with a concentration of 0.72 mol/L and a pH of 5.0 was introduced to the solution. A magnetic stirrer was used to stir the mixture using a water bath (40 °C) for a duration of 4 h. Upon the completion of the reaction, the sample was titrated with a 0.5 mol/L NaOH solution until the pH returned to 5.0. The aldehyde group content (AC) in the OxP molecules was determined according to the following formula.

AC (mmol/g) = (Vc − Vb) × C_NaOH_/m

Among them, Vc refers to the volume of the NaOH solution consumed in titrating the OxP solution. Vb refers to the volume of the NaOH solution consumed by the pullulan solution. C_NaOH_ (mol/L) refers to the concentration of the NaOH solution and m (g) refers to the mass of pullulan.

### 5.3. Cytotoxicity Test of the OxP Crosslinked Gelatin Hydrogels

After the hydrogels were individually placed into a freeze-dryer and subjected to a 24 h drying period, six lots of 5 mg gels, each with varying concentrations of OxP, were weighed and sterilized for further use. The gels from each group were then immersed in 5 mL of DMEM/F12 medium containing 10% FBS and 1% penicillin–streptomycin and incubated at 37 °C for 24 h to obtain an extract. ASCs, separated as per our previous studies in passage 3, were subsequently inoculated into a 96-well plate, with each well containing 5000 cells [11,12,41]. Six multiple wells were established for each group, and the cells were cultured for 24 h, denoted as OD_t_. Next, the supernatant was aspirated, followed by the gradual addition of culture medium containing CCK-8 (CCK-8: DMEM/F12 culture medium = 1:9). The resulting mixture was then placed in a cell culture incubator and incubated for 2 h. Subsequently, a microplate reader was employed to measure the absorbance value at a wavelength of 450 nm. Cytotoxicity can be derived from the obtained optical density (OD) value according to the following formula.

OD=ODt−ODbcODc−ODbc

where, OD_c_ and OD_bc_ refer to the optical densities of the control group (culture medium and cells) and blank control group (culture medium), respectively.

### 5.4. WHC and Degradation Rate of the Double-Crosslinked Hydrogels

The OxP and CaCl_2_ double-crosslinked gelatin–alginate hydrogel was placed in individual wells of a 48-well plate, with each well containing 1 mL of the hydrogel. Subsequently, 0.5 mL of a 3% (*w*/*v*) CaCl_2_ solution was added to each well. The plate was then placed in a 37 °C incubator for 30 min. After 12 h, the hydrogels were weighted. After the hydrogels were subjected to a freeze-drying for a period of 24 h, another weight measurement was carried out. The WHCs of the hydrogels were determined using the following formula:

$$\mathrm{WHC}=(W_{1}-W_{2})/W_{1}\times 100\%$$

where W_1_ and W_2_ refer to the wet and dry weights, respectively.

For the degradation rate (DR) measurement, the hydrogels with varying proportions were firstly immersed in PBS solution and incubated at 37 °C for 12 h before being frozen and dried for an additional 12 h, following which, their weights were measured as W1. The hydrogels were then re-immersed in PBS solution for consecutive intervals of 3, 6, 9, 12, and 15 d. Following each immersion period, the freeze-drying process was repeated, and the resulting dry weight was recorded as W2. The DR of the single- or double-crosslinked hydrogels was determined using the following calculation:

$$\mathrm{DR}=(W_{1}-W_{2})/W_{1}\times 100\%$$

### 5.5. Micropore Structure Characterization

The inner micropore structure of the hydrogels after dehydration was characterized using scanning electron microscopy (SEM). Briefly, after the samples were subjected to 30 s of immersion in liquid nitrogen, they were precisely cut into sections using a surgical blade. The sections underwent gold sputter coating and were examined under SEM for the purpose of microstructural analysis.

### 5.6. Induction of the ASCs into Endothelial Cells

According to our previous reports, bFGF and VEGF were used to stimulate the differentiation of the ASCs into endothelial cells [42,43]. Here, the cell induction solution (or medium) was prepared according to Table 2.

Induction steps: (1) weighting 1000 ng of VEGF and 200 ng of b-FGF, respectively, and adding them to 20 mL of DMEM/F-12 culture medium (containing 2% FBS and 1% penicillin–streptomycin) to obtain a cell induction medium; and (2) taking the passage 3 generation ASCs for the experiments. When the ASCs grew to about 85% confluence, they were cultured with the cell induction medium. The induction medium was changed every 48 h for a total of 7 d of induction.

### 5.7. Identification of the Endothelial-Induced ASCs

Immunofluorescence and Western blot techniques were used to detect the expression of endothelial cell marker CD31.

Immunofluorescence steps: after 7 d of induction, the cells were collected, fixed with paraformaldehyde, and blocked with goat serum for 30 min. Primary antibody (ABclonal, Wuhan, China, CD31Rabbit, 1:500) was added to the samples before they were incubated at 4 °C overnight. Then, a secondary antibody (APEXBIO, Goat Anti-rabbit IgG/AlexaFluro555, 1:200) was added dropwise before the samples were incubated at 37 °C in the dark for 1 h. After the samples were stained with DAPI, they were placed under a fluorescence microscope for observation.

Western blot steps: after 7 d of induction, the cells were collected and placed in a 105 °C metal bath for 5 min to obtain the protein-containing samples. The samples were analyzed via an 8% SDS-PAGE electrophoresis gel, beginning at a voltage of 80 V and completing at 120 V. After 2 h of transfer at 100 V, the protein strips were blocked with 5% skimmed milk powder for 1 h. The primary antibodies CD31 (ABclonal, CD31 Rabbit, 1:1000) and β-actin (ABclonal, 1:1000) were added to the samples before they were placed in a shaker at 4 °C overnight. A corresponding secondary antibody (1:20,000) was then employed before the samples were incubated at room temperature for 1.5 h, washed with TBST, stained with luminescent liquid, and inspected using a fluorescence imager. 

### 5.8. Cell Migration Test

HUVECs were chosen to assess the migratory capability of the cells cultured with the double-crosslinked hydrogel extraction media. The cells, extracts of G-A and G-A-2% OxP hydrogels, and a control (i.e., standard culture medium) were prepared accordingly. Once the cells, cultured in a 6-well plate, reached approximately 90% confluence, they were gently scraped using the tip of a sterile 200 μL pipette to make uniform scratches. The culture medium was then replaced with the extraction media supplemented with 2% FBS. Subsequently, images were captured using an inverted microscope at 0, 6, and 24 h after scratching, and these images were analyzed using Image J software (version 1.8.0). The closure area of the scratches was determined by measuring the distance of cell migration, and the resulting data are expressed as a relative percentage of the initial scratch size.

### 5.9. Printability and Biocompatibility of the Hydrogels

After a mixture with the final concentration of 5% (*w*/*v*), gelatin—3% (*w*/*v*), and sodium alginate—2% (*w*/*v*) OxP was prepared, it was transferred into the 3D printing barrel. The printing parameters were set up, including a layer height of 0.15 mm, contour filling once, a grid filling degree of 1.5, a printing speed of 3 mm/s, a printing angle ranging from 0 to 90°, a platform temperature of 4 °C, and manual regulated printing air pressure. A computer-aided design (CAD) model, such as a hollow cylinder, cuboid, five-pointed star, and kidney, was established using Solidworks. The 3D printing was carried out according to the predetermined CAD models, followed by crosslinking the 3D objects with a 3% (*w*/*v*) CaCl_2_ solution.

The biocompatibility of the cells in the 3D-printed gelatin–alginate hydrogels was characterized through AO/PI staining. Briefly, the gelatin–alginate-OxP solution (i.e., 5%, gelatin—3%, alginate—2% OxP) was firstly sterilized at 75 °C for 1 h, before the temperature was decreased to 37 °C and ASCs were added with a density of 5 × 10^5^ cells/mL. After 3D printing, the samples were put into a 6-well plate, followed by the addition of 5 mL of DMEM/F-12 complete culture medium to the wells and culturing in a 37 °C, 5% CO_2_ incubator. After 1, 3, and 14 d of culture, the samples were washed thrice with PBS buffer and stained with 1 mL of AO/PI dye solution for 15 min. Following this, the samples were washed again with PBS solution before being examined with an inverted fluorescence microscope.

### 5.10. Coaxially 3D Printing Vascular Networks

A micro syringe pump was used to install the coaxial 3D printing instrument. The cell density of the ASCs and HUVECs in the gelatin–alginate–OxP solution (i.e., 5% gelatin—3% alginate—2% OxP) was 5 × 10^5^ cells/mL.

There were several parameters that directly influenced the wall thickness and porosity of the 3D-printed vascular networks. One of the parameters was the extrusion velocities of the inner and outer shaft syringe pumps. The optimized parameters for the coaxial 3D printing nozzle are shown in Table 3. This means that the inner shaft used 3% CaCl_2_ solution with an extrusion speed of 500 μL/min, meanwhile the outer shaft used the gelatin–alginate–OxP solution as the bioink with an extrusion rate of 400–700 μL/min. Post-processing, the 3D objects were cut into sections and checked under a stereomicroscope. The obtained images were then analyzed using ImageJ to calculate the inner diameter, outer diameter, and wall thickness of the vascular networks. Statistical analysis was performed using Prism 8.0.2 software.

### 5.11. Histological Characterization of the Cell-Containing Vascular Networks

After 3D printing, the tubular vascular networks were cultured for a duration of three days, followed by freeze-drying and subsequent staining with DAPI, as stated above. The stained samples were observed using Olympus Fluorescence microscopy.

For HE staining, the cut samples were immersed in PBS buffer for a duration of 10 min after rewarming. Following this step, the samples were exposed to a hematoxylin staining solution for a period of 4 min and rinsed with tap water three times. A differentiation solution was used for a duration of 3 s, followed by another three rinses with tap water. After the blue solution was restored for a duration of 40 s and subsequently soaked in tap water for 30 s, eosin staining was performed for a duration of 1 min, followed by a 20 s soak in tap water. The samples were finally watched under an upright microscope after being dehydrated and sealed with neutral gum. 

### 5.12. Construction of the Vascularized Tissues

The design of the vascularized tissues with go-through channels was accomplished using SolidWorks software (SolidWorks 2021), followed by the construction of the physical CAD model utilizing extrusion-based 3D printing technologies. Upon the completion of the 3D printing process, the 3D-printed constructs were crosslinked using a 3% (*w*/*v*) CaCl_2_ solution before they were cultured and examined. 

### 5.13. Phalloidin Staining and Vascular Connectivity Testing

It has been reported that fluorescently labeled phalloidin exhibits a specific affinity towards F-actins, which led to the fluorescence labelling of the cytoskeleton and visualization of the cellular growth dynamics within the 3D constructs [44].

AbFluor 594-labeled Phalloidin was employed for the purpose of staining the coaxially printed vascular networks, thereby facilitating enhanced observation of the growth status of the HUVECs within the vascular networks. The procedure entailed, after the 3D-printed constructs were put into a 6-well plate and stained with phalloidin, fixing the samples with 4% paraformaldehyde and washing them three times with PBS buffer supplemented with 0.1% TritonX-100. AbFluor594-labeled Phalloidin (Abbkine) was then added in the dark and incubated at room temperature for 1 h. A DAPI dye solution was then added dropwise and incubated for a period of 5 min. After removing the DAPI solution, the samples were washed again, with each wash lasting 5 min.

To assess the connectivity of the vascular network, a 1 mL syringe was employed to administer a gradual injection of a 25 μg/mL FITC-conjugated 40 kDa dextran solution into a specific segment of the vascular network. Upon the solution’s emergence from the opposite end, a hamostatic clip was utilized to secure the two sections. Subsequently, PBS was employed for further rinsing to eliminate any residual fluorescent solution at the external end. Finally, the samples were observed using an inverted fluorescence microscope.

## Figures and Tables

**Table 1:** Polymeric components in the gelatin–alginate hydrogels.

Group	1	2	3	4	5
Gelatin (%, *w*/*v*)	5	5	5	5	5
Sodium alginate (%, *w*/*v*)	3	3	3	3	3
OxP (%, *w*/*v*)	0	0.5	1	1.5	2

**Table 2:** Endothelial cell growth factor concentration.

Growth Factor	Concentration (ng/mL)
Rat vascular endothelial growth factor (VEGF)	50
Basic fibroblast growth factor (b-FGF)	10

**Table 3:** Extrusion speed and flow rate ratio of the outer/inner shaft flows.

Group	1	2	3	4	5	6
Outer shaft flow velocity (μL/min)	400	450	500	550	600	700
Inner shaft flow velocity (μL/min)	500	500	500	500	500	500
Flow rate ratio (outside: inside)	0.8	0.9	1	1.1	1.2	1.4

## Data Availability

The datasets used and analyzed during the current study are available online.
